# The influence of caregiver type on isolation distress and care satisfaction in hematopoietic stem cell transplant patients: the mediating role of relationships

**DOI:** 10.3332/ecancer.2026.2155

**Published:** 2026-06-25

**Authors:** Anjali Rathee, Priyanshi Dixit, Surya Kant Tiwari, Mukul Aggarwal, Pradeep Kumar, Rishi Dhawan, Richa Chauhan, Jasmita Dass, Ganesh Kumar Vishwanathan, Tulika Seth, Manoranjan Mahapatra

**Affiliations:** 1 Department of Nursing Services, All India Institute of Medical Sciences, New Delhi 110029, India; 2 College of Nursing, VMMC and Safdarjung Hospital, New Delhi 110029, India; 3 College of Nursing, All India Institute of Medical Sciences, Raebareli, Uttar Pradesh 229405, India; 4 Department of Hematology, All India Institute of Medical Sciences, New Delhi 110029, India; a https://orcid.org/0009-0004-0460-4917; b https://orcid.org/0000-0001-5130-4037; c https://orcid.org/0000-0003-4718-0398

**Keywords:** hematopoietic stem cell transplantation, protective isolation, isolation distress, care satisfaction, caregiver type, relationship quality, mediation analysis

## Abstract

**Background:**

Protective isolation is essential for preventing infection in patients undergoing hematopoietic stem cell transplantation (HSCT). However, it is associated with significant psychosocial morbidity. Understanding the factors influencing isolation perception and its impact on care satisfaction is crucial, particularly in resource-limited settings.

**Methods:**

This cross-sectional observational study enrolled 47 adults who underwent autologous or allogeneic HSCT at a tertiary care center in India. Perceptions of isolation and satisfaction with care were measured using validated ISOLA and FAMCARE-P16 scales, respectively. Data were analysed using correlation, multivariate regression and mediation analyses.

**Results:**

Cluster analysis revealed that nearly half of the patients experienced a high-isolation (48.9%) and low-satisfaction (46.8%) profile. Multivariate regression identified that having a non-partner caregiver was the strongest predictor of higher perceived isolation (B = 5.62, p = 0.002). Regarding satisfaction, being unmarried was the most significant predictor of lower satisfaction (B = −24.66, p < 0.001), followed by non-Hindu religion, allogeneic transplant and poorer relationships with others. Critically, mediation analysis revealed that the effect of caregiver type on satisfaction was fully mediated by the patient’s perceived relationship with others (indirect effect: −2.71, 95% confidence intervals: −6.22 to −0.41).

**Conclusion:**

A significant proportion of HSCT recipients experience a detrimental combination of high isolation and low satisfaction. In this exploratory study, the finding that a partner caregiver may improve satisfaction by mitigating relational alienation highlights a modifiable psychosocial target. Clinical strategies should focus on screening high-risk patients and implementing interventions to strengthen social connections for those without partners or caregivers; however, larger studies are needed to confirm these findings.

## Introduction

Hematopoietic stem cell transplantation (HSCT) is a potentially curative intervention for an expanding range of haematological malignancies and non-malignant disorders [1, 2]. This procedure involves the administration of myeloablative chemotherapy or radiotherapy, followed by the infusion of healthy hematopoietic stem cells to reconstitute the immune system [3]. Although life-saving, this process induces profound pancytopenia, rendering patients exceptionally vulnerable to life-threatening infections [4, 5].

To mitigate this risk, protective isolation, including strict hand hygiene, protective gear for visitors and placement in a specialised positive-pressure room, remains a cornerstone of standard care during the neutropenic phase [6, 7]. The clinical efficacy of these measures in reducing exogenous infections has been well-documented. However, the psychological sequelae of this necessary confinement are increasingly recognised as critical aspects of the patient experience. This experience, characterised by sensory deprivation, limited social contact and loss of autonomy, can precipitate significant psychosocial morbidity, including loneliness, anxiety, depression and diminished quality of life (QoL) [8, 9].

A growing body of evidence from high-income countries has begun characterising this burden [9, 10]. Patients report feelings of boredom, imprisonment and profound disconnection from their support systems, which can compound the physical suffering from treatment-related toxicities, such as oral mucositis and graft-versus-host disease [8, 11]. Furthermore, a patient’s perception of their care during this vulnerable period is a critical determinant of their overall experience. Satisfaction with care, defined as the alignment between patient expectations and the reality of care received, is intrinsically linked to better adherence, improved trust in providers and may even influence clinical outcomes [12, 13]. The key domains influencing satisfaction include the provision of clear information, effective communication and empathetic support [14].

Crucially, the experience of isolation is not uniform and is likely to be moderated by a complex interplay of sociodemographic, clinical and cultural factors. For instance, educational attainment may influence coping mechanisms [15], whereas the nature of the caregiver relationship can buffer emotional distress [16]. However, the existing literature has some limitations. First, studies have predominantly focused on Western populations, and the cultural context of countries like India, with its strong familial structures but often overburdened healthcare systems, may profoundly shape the experience of isolation [17]. Second, the relationship between specific dimensions of isolation-related distress and overall satisfaction with care remains underexplored, particularly in real-world clinical settings.

Therefore, a critical gap exists in understanding the psychosocial landscape of HSCT patients undergoing protective isolation in low- and middle-income country (LMIC) settings, particularly the mechanisms through which isolation influences patient-centered outcomes, such as satisfaction with care. Understanding this pathway is essential for developing targeted interventions to improve patient experiences in LMIC settings. This study aimed to address this gap by cross-sectionally evaluating the perceptions of protective isolation and pathways to satisfaction among patients undergoing HSCT at a tertiary care center in India.

Our primary objectives were to (1) identify the sociodemographic and clinical factors associated with perceived isolation, (2) examine the association between distinct domains of isolation and patient satisfaction with care and (3) explore potential mediating pathways linking these experiences.

## Material and methods

### Study design and setting

This cross-sectional observational study was conducted in the bone marrow transplantation unit of a tertiary care hospital in northern India. The study utilised a consecutive sampling approach, in which all eligible patients admitted for HSCT between November 2023 and June 2025 were invited to participate.

### Participants

Eligible participants were adults (age ≥18 years) undergoing either autologous or allogeneic HSCT who could comprehend and complete the study questionnaires in Hindi or English. Patients were excluded if they had cognitive impairment or were medically unstable at the time of assessment, as determined by the treatment team.

### Sample size

A formal sample size calculation was not conducted a priori because of the exploratory nature of the study and the finite population of eligible patients undergoing HSCT during the designated period. The final sample comprised 47 consecutive patients who met all the eligibility criteria and provided written informed consent.

### Variables and data sources

Demographic and clinical variables, including age, sex, education level, marital status, caregiving arrangement, primary diagnosis and transplant type (autologous versus allogeneic), were extracted from medical records and supplemented by interviews with the patients. The clinical complications were defined as follows:

**Fever:** A single oral temperature ≥38.3°C or a sustained temperature ≥38.0°C for over 1 hour during the protective isolation period. For descriptive and analytic purposes, any recorded temperature ≥37°C was categorised as ‘elevated temperature’ to capture subclinical temperature variations during isolation.

**Oral mucositis:** Graded by the treating physician according to the World Health Organisation Oral Toxicity Scale [18].

### Outcome measures

The perception of protective isolation was assessed using the ISOLA scale, a validated instrument that evaluates three domains: isolation-related suffering, relationships with others and relationships with oneself [19]. Higher scores in each domain indicate greater isolation-related distress (i.e., poorer relationships, more suffering). The scale demonstrated excellent internal consistency in the present sample (Cronbach’s *α* = 0.95).

Satisfaction with care was measured using the FAMCARE-P16 tool [20], which assesses satisfaction across multiple domains of care, including the provision of medical information, emotional support and physical care. Although originally validated in outpatient advanced cancer populations [20], the FAMCARE-P16 assesses domains – provision of information, emotional support and physical care – that are central to the inpatient HSCT experience. Given the lack of a validated HSCT-specific satisfaction instrument, we deemed this to be the most appropriate available measure. Responses were recorded on a 5-point Likert scale (1 = ‘very dissatisfied’ to 5 = ‘very satisfied’), with higher scores indicating greater patient satisfaction. The measure also showed excellent internal consistency in this study (Cronbach’s *α* = 0.98).

### Data collection procedures

All questionnaires were completed by the patients at the bedside during the protective isolation period, with assistance from the investigator to minimise participant burden and ensure privacy. Missing or unclear responses were clarified immediately with the participant; therefore, no questionnaires were excluded due to incomplete data.

### Ethical considerations

The study protocol was approved by the Institutional Ethics Committee (Ref. No.: IEC-639/06.10.2023, RP-5/2023). Before enrolment, written informed consent was obtained from all participants after a detailed discussion of the study objectives and procedures. Participation was voluntary, and the participants were assured that their standard of care would not be affected if they chose to withdraw from the study. All collected data were anonymised using unique study identification numbers to ensure data confidentiality.

### Data analysis

Data were analysed using SPSS version 26.0 (IBM Corp., Armonk, NY, USA). Descriptive statistics are presented as frequencies and percentages for categorical variables and as means ± standard deviation for continuous variables. The internal consistency of the ISOLA and FAMCARE-P16 scales was assessed using Cronbach’s alpha. Two-step cluster analysis was employed to classify patients into natural groupings based on the total ISOLA score and, separately, the total FAMCARE score. The optimal number of clusters was determined automatically by the algorithm based on the log-likelihood distance measure and Schwarz’s Bayesian Information Criterion. Bivariate relationships between perceived isolation domains and satisfaction with care were quantified using Pearson’s correlation coefficient (*r*).

Multiple linear regression analyses were used to identify the independent predictors of perceived isolation and satisfaction with care. All variables with a significance level of *p* < 0.20 in the univariate analyses were considered for inclusion in the initial multivariate model. The models were constructed using the enter method. Multicollinearity was assessed using the variance inflation factor (VIF); variables with a VIF >5 were considered to have high multicollinearity and were excluded from the final model. The overall significance of each regression model was assessed using an *F*-test, and the proportion of variance explained was reported as the adjusted *R*² value.

A mediation analysis was conducted using the PROCESS macro for SPSS (version 4.2) to test the hypothesis that the relationship between caregiver type and satisfaction was mediated by the patient’s relationship with others. The significance of the indirect effect was determined using bias-corrected bootstrap confidence intervals (CIs) (based on 5,000 samples), where a 95% CI not containing zero was considered significant, as this method is robust to non-normality in small sample sizes. For all analyses, a two-tailed *p*-value of <0.05 was considered statistically significant.

## Results

### Participant characteristics

A total of 47 patients who underwent HSCT were enrolled in this study. The socio-demographic and clinical characteristics of the participants are presented in Table 1. The mean age was 37.68 ± 14.88 years, with the majority being male (63.8%), educated below secondary school (63.8%) and married (61.7%). Most patients identified as Hindu (68.1%) and had a non-partner (74.5%) as their primary caregiver. The most common diagnosis was acute myeloid leukaemia (40.4%). More than half of the patients underwent allogeneic transplantation (57.4% of patients). During the assessment period, 34.0% of patients had an elevated body temperature (≥37°C) and 25.5% had moderate-to-severe oral mucositis (Grade 2–3).

### Perceptions of isolation and satisfaction with care

Separate two-step cluster analyses were performed for the total ISOLA and FAMCARE-P16 scores to classify patients into high- and low-scoring groups. For perceived isolation, the analysis revealed two distinct clusters: Low isolation (*n* = 24, 51.1%) and high isolation (*n* = 23, 48.9%). For satisfaction with care, the clusters were classified as low satisfaction (*n* = 22, 46.8%) and high satisfaction (*n* = 25, 53.2%) (Table 2).

Table 3 presents the bivariate correlations between the variables of this study. The total perceived isolation score was strongly and positively correlated with all its subscales (isolation-related suffering, relationship with others and relationship with oneself; *p* < 0.01). As hypothesised, a higher total perceived isolation score was significantly correlated with lower satisfaction with care (*r* = −0.344, *p* < 0.01). Among the subscales, poor relationships with others (*r* = −0.364, *p* < 0.05) and greater isolation-related suffering (*r* = −0.339, *p* < 0.05) were significantly and negatively correlated with satisfaction.

### Factors associated with perceived isolation and satisfaction

Multiple linear regression analyses were used to identify the independent predictors of outcomes. Variables with a univariate *p*-value <0.2 were incorporated into the initial models.

The model for perceived isolation was statistically significant (*F* = 7.154, *p* < 0.001) and explained 40.1% of the variance (adjusted *R*² = 0.401). As shown in Table 4, after entering age, marital status, carer, religion and temperature, the type of caregiver emerged as the only significant independent predictor (*B* = 5.624, *β* = 0.458, *p* = 0.002). Patients without partner caregivers reported significantly higher levels of perceived isolation. Multicollinearity diagnostics indicated acceptable VIF values for all retained predictors (all VIF < 3). Specifically, the VIF values for marital status and caregiver type were 2.72 and 1.52, respectively, indicating no problematic collinearity.

The model for satisfaction with care was also highly significant (*F* = 13.133, *p* < 0.001), explaining 61.3% of the variance (adjusted *R*² = 0.613). The initial independent variables were age, education, marital status, religion, type of HSCT and three ISOLA subscales. Age and isolation-related suffering were removed from the final model because the VIF values exceeded 5, indicating multicollinearity. In the final model, marital status was the strongest predictor (*B* = −24.66, *β* = −0.83, *p* < 0.001), with unmarried patients reporting significantly lower levels of satisfaction. Furthermore, having a non-Hindu religion (*B* = 9.29, *β* = 0.30, *p* = 0.027), undergoing allogeneic transplantation (*B* = −8.31, *β* = −0.28, *p* = 0.024) and reporting a poor relationship with others (*B* = −2.89, *β* = −0.30, *p* = 0.044) were independently associated with lower satisfaction with care.

## Mediating role of relationship with others

A mediation analysis was conducted to understand the mechanism linking caregiver type and satisfaction with care. We tested a model in which the effect of caregiver type (X) on satisfaction with care (Y) was mediated by the patient’s perceived relationship with others (M). The results are presented in Table 5.

Path analysis revealed a significant positive effect of having a partner caregiver on relationships with others (Path a: *B* = 0.78, *p* < 0.001). Note that higher scores on the ISOLA ‘Relationship with others’ subscale denote poorer perceived relationships; thus, the positive coefficient for Path a indicates that having a non-partner caregiver is associated with poorer relational quality. In turn, a poorer relationship with others had a significant negative effect on satisfaction with care (path b: *B* = −3.48, *p* = 0.017). Because the ISOLA scale is coded such that higher values reflect greater distress, this negative coefficient confirms that worsening relationships predict declining relationship satisfaction. The direct effect of caregiver type on satisfaction was non-significant (Path c’: *B* = −0.033, *p* = 0.990). Most importantly, the bootstrap CI for the indirect effect (a*b) did not include zero (*B* = −2.71, 95% CI: −6.22, −0.41), confirming a significant mediating effect, despite the non-significant normal theory *p*-value (*p* = 0.062). The negative estimate for the indirect effect indicates that having a non-partner caregiver (coded higher than a partner) is associated with a decrease in satisfaction through its negative effect on relationships with others. This suggests that the beneficial effect of having a partner caregiver on satisfaction is fully mediated by an improved perceived relationship with others during protective isolation.

## Discussion

This prospective study sheds light on the complex psychosocial dynamics of protective isolation and its critical link with satisfaction with care among HSCT recipients. While isolation is a recognised source of distress, our findings advance the field by identifying a high-risk patient profile and, most significantly, unveiling the mechanistic pathway through which the social environment shapes the care experience. We demonstrate that the absence of a partner caregiver is a primary driver of isolation distress, an effect that subsequently undermines satisfaction with care through the mediator of relational alienation.

The significant burden of protective isolation is clearly illustrated by our cluster analysis, which found that nearly half of the patients endured a high-isolation, low-satisfaction trajectory. This aligns with the prior work by Biagioli *et al* [21] who documented moderate-to-severe suffering across the isolation domain. Our correlational analysis further confirmed the interwoven nature of this experience, showing that feelings of isolation are intrinsically linked to worsened relationships with others and oneself. This constellation of loneliness, anxiety and depression is a well-documented sequelae of HSCT, often exacerbated by pre-existing personal or familial stressors [22–25]. This reinforces the critical need for pre-transplant psychological screening to identify vulnerable individuals, allowing for proactive support before the isolation period begins [26].

A pivotal and novel finding of our study is that having a non-partner caregiver emerged as the strongest independent predictor of perceived isolation. This finding adds a crucial layer of specificity to the literature. While some studies have found no direct link between caregiver type and isolation [21], others emphasise that a close caregiver can provide essential support and foster a sense of belonging [27, 28]. Our results suggest that, in the context of profound isolation, a partner may offer a unique and irreplaceable form of emotional intimacy and advocacy that cannot be replicated by other family members. This is not to diminish the role of other caregivers but to highlight potential vulnerabilities. The demands of caregiving can also isolate caregivers, creating a reciprocal cycle of distress [29]. Therefore, a paradigm shift is needed: caregiver evaluation and support through counselling and targeted sessions must become a standard component of pre-transplant care to safeguard the well-being of this vital dyad.

Regarding satisfaction with care, our regression model revealed that being unmarried was the most powerful predictor of lower satisfaction, consistent with studies highlighting the role of a spouse as a primary advocate and source of security within the healthcare system [30]. Most importantly, we found that poor relationships with others were key independent predictors of dissatisfaction. This indicates that a feeling of relational disconnect directly corrodes the patient’s perception of the care received. This underscores the importance of healthcare providers employing a human-connecting, communicative approach that involves the family in decision-making [21, 31–33]. However, our study also reflects a paradox noted in the literature: some patients intentionally withdraw from communication to focus on their recovery [22, 31, 34]. This suggests that the quality and patient-desired level of interaction are more critical than the quantity, moving beyond a one-size-fits-all approach to providing psychosocial support.

The culmination of our analysis is a mediation model that provides a groundbreaking mechanistic explanation for these observations. We found that partner caregivers’ protective effect on satisfaction was fully mediated by patients’ perceived relationships with others. This is a paradigm-shifting insight. This demonstrates that the benefit of a partner is not merely their presence but their unique efficacy in maintaining the patient’s sense of relational connectedness, thus buffering them against the alienating effects of isolation. Consequently, the patient’s evaluation of care becomes less about technical proficiency and more about whether their fundamental human need for connection is being met. This finding mandates that interventions aimed at improving satisfaction must extend beyond clinical care to actively facilitate and strengthen social connections, especially among patients without partners or caregivers.

### Strengths

This study had several strengths. To the best of our knowledge, this is the first study to cross-sectionally examine the psychosocial experience of protective isolation in an Indian setting, addressing a significant gap in LMIC. The use of validated patient-reported outcome measures (ISOLA and FAMCARE-P16) ensured robust capture of the subjective patient experience. The application of advanced statistical methods, including multivariate regression and mediation analysis, moves beyond descriptive findings to identify independent predictors and, crucially, the mechanistic pathway linking caregiver type and satisfaction. This mechanistic insight is a key conceptual strength that provides a clear target for future studies.

### Limitations

The interpretative power of these findings must be considered in light of the study’s limitations. The single-center design and modest sample size may affect the generalisability of the findings and limit the statistical power to detect smaller effect sizes. The cross-sectional nature of the assessment precludes definitive causal inference. Furthermore, the modest sample size limits the statistical power of the mediation analysis; thus, the findings should be considered preliminary and require replication in larger cohorts. Although validated instruments were used, reliance on self-reported measures introduces the potential for recall and social desirability bias, particularly as an investigator was present to assist with questionnaire completion. Future studies should employ self-administered electronic surveys to minimise this effect. Furthermore, unmeasured confounding variables, such as premorbid psychological health, specific coping strategies and the quality of the relationship between the patient and caregiver, may influence outcomes and should be investigated in future studies.

### Practical implications

The findings of this study have direct and actionable clinical implications. First, upon admission, patients should be screened for psychosocial vulnerability, with specific attention to those without a partner caregiver and unmarried individuals, as they constitute a high-risk group for isolation, distress and dissatisfaction. Second, the pivotal role of caregivers must be formally recognised and supported; healthcare institutions should offer targeted educational and counselling sessions to prepare all caregivers for the challenges of isolation and equip them with strategies to provide effective and emotional support. Third, nursing and medical staff should be trained on the profound impact of relational health on patient outcomes. Protocols should encourage staff to act as ‘connection facilitators,’ dedicating time to meaningful interactions and leveraging technology to help patients maintain links with their broader social networks. Finally, structured pre-transplant education programs should explicitly address the psychological challenges of isolation and prepare patients and caregivers with evidence-based coping strategies to mitigate this distress.

## Conclusion

In conclusion, this study demonstrates that the experience of protective isolation and perception of care is profoundly shaped by psychosocial factors. We identified patients without a partner caregiver as a vulnerable population at significant risk of severe isolation distress and subsequent care dissatisfaction. The novel finding that this effect is fully mediated by patients’ perceived relationship with others provides a clear and modifiable target for future interventions. This underscores that satisfaction is not merely a function of clinical technicalities but is deeply intertwined with emotional and social well-being. Therefore, a paradigm shift towards holistic, psychosocially informed supportive care is essential. Future research should validate these findings in larger longitudinal cohorts and develop targeted interventions to replicate the protective ‘partner effect,’ thereby humanising protective isolation and improving the QoL of all HSCT recipients.

## Conflicts of interest

The authors declare no conflicts of interest.

## Funding

This research did not receive any specific grants from funding agencies in the public, commercial or not-for-profit sectors.

## Figures and Tables

**Table 1.** Socio-demographic and clinical characteristics of participants (n = 47).

Variables	Category	Frequency (%)
Age*		37.68 ± 14.88 (19–65)
Age group (years)	<35 >35	22 (46.8) 25 (53.2)
Sex	Male	30 (63.8)
	Female	17 (36.2)
Education	Below secondary school	30 (63.8)
	Secondary school or above	17 (36.2)
Marital status	Married	29 (61.7)
	Unmarried	18 (38.3)
Religion	Hindu	32 (68.1)
	Others	15 (31.9)
Carer	Partner	12 (25.5)
	Non-partner (parent, sibling or child)	35 (74.5)
Diagnosis	AML	19 (40.4)
	MM	13 (27.7)
	NHL	7 (14.9)
	ALL	3 (6.4)
	MDS	5 (10.6)
Type of PBSCT	Autologous	20 (42.6)
	Allogeneic	27 (57.4)
Body temperature (°C)	<37	31 (66.0)
	≥37	16 (34.0)
Day post–transplant	+7	26 (55.3)
	+8	18 (38.3)
	+9	3 (6.4)
Oral mucositis	Grade 0	18 (38.3)
	Grade 1	17 (36.2)
	Grade 2	11 (23.4)
	Grade 3	1 (2.1)
* Mean ± SD (Range) **Abbreviations:** AML, acute myeloid leukaemia; MM, multiple myeloma; NHL, non-Hodgkin lymphoma; ALL, acute lymphoblastic leukaemia; MDS, myelodysplastic syndrome		

**Table 2.** Two-step cluster analysis of total ISOLA and FAMCARE scores (n = 47).

Variables	Frequency (%)
Perceived isolation Low High	24 (51.1%) 23 (48.9%)
Satisfaction with care Low High	22 (46.8) 25 (53.2)

**Table 3.** Correlations between perceived protective isolation and satisfaction with care.

	1	2	3	4	5
Perceived isolation (1)	1	0.987**	0.856**	0.920**	−0.344**
Isolation–related suffering (2)		1	0.800**	0.861**	−0.339*
Relationship with others (3)			1	0.756**	−0.364*
Relationship with oneself (4)				1	−0.274
Satisfaction with care (5)					1
** correlation is significant at the 0.01 level (2-tailed) * correlation is significant at the 0.05 level (2-tailed)					

**Table 4.** Multiple linear regression analysis of the factors associated with perceived isolation and satisfaction with care.

	B	Standard error	β	t	p	95% CI for B
Perceived isolation						
Constant	26.208	12.038	-	2.177	0.035	1.897, 50.520
Age	−0.024	0.137	−0.033	−0.174	0.863	−0.300, 0.252
Marital status	4.116	4.141	0.187	0.994	0.326	−4.246, 12.478
Carer	5.624	1.729	0.458	3.253	0.002**	2.132, 9.116
Religion	0.840	3.223	0.037	0.261	0.796	−5.669, 7.349
Elevated temperature	−4.545	2.760	−0.201	−1.647	0.107	−10.120, 1.029
Satisfaction with care						
Constant	100.295	8.746	-	11.468	<0.001**	82.619, 117.970
Education	−2.633	3.115	−0.087	−0.845	0.403	−8.930, 3.663
Marital status	−24.659	3.421	−0.827	−7.208	<0.001**	−31.574, −17.745
Religion	9.291	4.057	0.299	2.290	0.027*	1.090, 17.491
Type of HSCT	−8.311	3.555	−0.284	−2.338	0.024*	−15.495, −1.127
Relationship with others	−2.891	1.391	−0.302	−2.079	0.044*	−5.702, −0.080
Relationship with oneself	1.420	0.877	0.246	1.620	0.113	−0.352, 3.193
**Note:** *B* = Unstandardised coefficient; *β* = Standardised coefficient. For the perceived isolation model: *R*² = 0.466, Adjusted *R*² = 0.401, F(5,41) = 7.154, *p* < 0.001. For satisfaction model: *R*² = 0.658, Adjusted *R*² = 0.613, F(6,40) = 13.133, *p* < 0.001. Multicollinearity diagnostics indicated acceptable VIF values for all the retained predictors (all VIF < 3.0). * *p* < 0.05; ** *p* < 0.01						

**Table 5.** Mediation analysis testing relationship with others as a mediator between caregiver type and satisfaction with care.

Path	Estimate	SE	Z	p-value	95% bootstrap CI
Direct effects					
Carer → Satisfaction (c')	−0.033	2.704	−0.012	0.990	−5.04, 5.59
Path a					
Carer → Relationship with others	0.777	0.178	4.375	< .001**	0.44, 1.12
Path b					
Relationship with others → Satisfaction	−3.483	1.466	−2.376	0.017*	−6.38, -0.64
Indirect Effect (a*b)					
Carer → Relationship with others → Satisfaction	−2.708	1.452	−1.865	0.062 †	−6.22, −0.41
† The normal theory *p*-value (Sobel test) was 0.062. However, the significance of the indirect effect was determined by the bias-corrected bootstrap 95% CI, which did not include zero, confirming a statistically significant mediation effect **Note:** Higher scores on the ISOLA ‘Relationship with others’ subscale indicate poorer perceived relationships. Therefore, the positive coefficient for Path a indicates that having a non-partner caregiver (coded higher) is associated with poorer relationship quality. The negative coefficient for path b confirmed that poorer relationships predicted lower satisfaction with care *p < 0.05; ** *p* < 0.01					

## References

[1] Khaddour K, Hana CK, Mewawalla P (2025). Hematopoietic Stem Cell Transplantation [Internet].

[2] Klein OR, Bonfim C, Abraham A (2023). Transplant for non-malignant disorders: an International Society for Cell & Gene Therapy Stem Cell Engineering Committee report on the role of alternative donors, stem cell sources and graft engineering. Cytotherapy.

[3] Galgano L, Hutt D HSCT: how does it work? [Internet]. The European Blood and Marrow Transplantation Textbook for Nurses: under the Auspices of EBMT 2018 eds M Kenyon and A Babic.

[4] Fu JB, Morishita S (2024). Inpatient rehabilitation of hematopoietic stem cell transplant patients: managing challenging impairments and medical fragility. Am J Phys Med Rehabil.

[5] Tomblyn M, Chiller T, Einsele H (2009). Guidelines for preventing infectious complications among hematopoietic cell transplantation recipients: a global perspective. Biol Blood Marrow Transplant.

[6] Soni S, Yarrarapu SNS, Guzman N (2025). Infection control. http://www.ncbi.nlm.nih.gov/books/NBK519017/.

[7] Siddharth V, Jamwal T, Aggarwal M (2024). Planning and designing of an inpatient isolation facility for hematopoietic stem cell transplant patients. Indian J Hematol Blood Transfus.

[8] Lee RS, Halliday LE (2025). The psychological effects of protective isolation on haematological stem cell transplant patients: an integrative, descriptive review. Support Care Cancer Off J Multinatl Assoc Support Care Cancer.

[9] Janicsák H, Ungvari GS, Gazdag G (2021). Psychosocial aspects of hematopoietic stem cell transplantation. World J Transplant.

[10] Amonoo HL, Massey CN, Freedman ME (2019). Psychological considerations in hematopoietic stem cell transplantation. Psychosomatics.

[11] Cooke L, Gemmill R, Kravits K (2009). Psychological consequences of hematopoieitc stem cell transplant. Semin Oncol Nurs.

[12] Ferreira DC, Vieira I, Pedro MI (2023). Patient satisfaction with healthcare services and the techniques used for its assessment: a systematic literature review and a bibliometric analysis. Healthcare.

[13] Donneyong MM, Bynum M, Kemavor A (2024). Patient satisfaction with the quality of care received is associated with adherence to antidepressant medications. PLoS One.

[14] Mularczyk-Tomczewska P, Gujski M, Koperdowska JM (2025). Factors influencing patient satisfaction with healthcare services in Poland. Med Sci Monit Int Med J Exp Clin Res.

[15] Corman M, Dambrun M, Rubio MT (2023). The prospective effects of coping strategies on mental health and resilience at five months after HSCT. Healthcare.

[16] Kim B, Hwang IC, Ahn HY (2025). Family relationships and caregiver burden among family caregivers of patients with terminal cancer. BMC Palliat Care.

[17] Chawla NS (2023). Unveiling the ABCs: identifying India’s Healthcare Service Gaps. Cureus.

[18] Lu K (2022). WHO Mucositis Scale.

[19] Biagioli V, Piredda M, Annibali O (2019). Development and initial validation of a questionnaire to assess patients’ perception of protective isolation following haematopoietic stem cell transplantation. Eur J Cancer Care (Engl).

[20] Lo C, Burman D, Hales S (2009). The FAMCARE-patient scale: measuring satisfaction with care of outpatients with advanced cancer. Eur J Cancer Oxf Engl 1990.

[21] Biagioli V, Piredda M, Annibali O (2019). Factors influencing the perception of protective isolation in patients undergoing haematopoietic stem cell transplantation: a multicentre prospective study. Eur J Cancer Care (Engl).

[22] Biagioli V, Piredda M, Alvaro R (2017). The experiences of protective isolation in patients undergoing bone marrow or haematopoietic stem cell transplantation: systematic review and metasynthesis. Eur J Cancer Care (Engl).

[23] Brice L, Gilroy N, Dyer G (2017). Haematopoietic stem cell transplantation survivorship and quality of life: is it a small world after all?. Support Care Cancer Off J Multinatl Assoc Support Care Cancer.

[24] Amonoo HL, Brown LA, Scheu CF (2020). Beyond depression, anxiety and post-traumatic stress disorder symptoms: qualitative study of negative emotional experiences in hematopoietic stem cell transplant patients. Eur J Cancer Care (Engl).

[25] Whittemore R, Knafl K (2005). The integrative review: updated methodology. J Adv Nurs.

[26] El‐Jawahri AR, Traeger LN, Kuzmuk K (2015). Quality of life and mood of patients and family caregivers during hospitalization for hematopoietic stem cell transplantation. Cancer.

[27] Gemmill R, Cooke L, Williams AC (2011). Informal caregivers of hematopoietic cell transplant patients: a review and recommendations for interventions and research. Cancer Nurs.

[28] Barata A, Tavori G, Wolff D (2024). Patient and physician communication in the allogeneic transplantation setting: challenges and potential solutions. Transplant Cell Ther.

[29] Sannes TS, Ranby KW, Yusufov M (2022). More often than not, we’re in sync: patient and caregiver well-being over time in stem cell transplantation. Health Qual Life Outcomes.

[30] Bahkali NM, Eissa GA, Alharbi FM (2022). Effect of premarital education on the quality of life of female partners: a cross-sectional study. Cureus.

[31] Annibali O, Pensieri C, Tomarchio V (2017). Protective isolation for patients with haematological malignancies: a pilot study investigating patients’ distress and use of time. Int J Hematol-Oncol Stem Cell Res.

[32] Cohen MZ, Ley C, Tarzian AJ (2001). Isolation in blood and marrow transplantation. West J Nurs Res.

[33] Williams BJ (2012). Self-transcendence in stem cell transplantation recipients: a phenomenologic inquiry. Oncol Nurs Forum.

[34] Dunn E, Arber A, Gallagher A (2016). The immediacy of illness and existential crisis: patients’ lived experience of under-going allogeneic stem cell transplantation for haematological malignancy. A phenomenological study. Eur J Oncol Nurs Off J Eur Oncol Nurs Soc.

